# How do we get there? Effects of cognitive aging on route memory

**DOI:** 10.3758/s13421-017-0763-7

**Published:** 2017-11-08

**Authors:** Mary O’Malley, Anthea Innes, Jan M. Wiener

**Affiliations:** 1 Department of Psychology, Ageing and Dementia Research Centre, Poole House, Talbot Campus, Fern Barrow, Bournemouth University, Poole, Dorset, BH12 5BB UK; 2 Faculty of Social Science, University of Stirling, Colin Bell Building, Stirling, FK9 4LA UK; 3 Salford Institute for Dementia, School of Nursing, Midwifery, Social Work and Social Sciences, University of Salford, Room 113d, Crescent House, Salford, M5 4WT UK

**Keywords:** Route learning, Navigation, Cognitive impairment, Spatial memory, Atypical aging

## Abstract

Research into the effects of cognitive aging on route navigation usually focuses on differences in learning performance. In contrast, we investigated age-related differences in route knowledge after successful route learning. One young and two groups of older adults categorized using different cut-off scores on the Montreal Cognitive Assessment (MoCA), were trained until they could correctly recall short routes. During the test phase, they were asked to recall the sequence in which landmarks were encountered (Landmark Sequence Task), the sequence of turns (Direction Sequence Task), the direction of turn at each landmark (Landmark Direction Task), and to identify the learned routes from a map perspective (Perspective Taking Task). Comparing the young participant group with the older group that scored high on the MoCA, we found effects of typical aging in learning performance and in the Direction Sequence Task. Comparing the two older groups, we found effects of early signs of atypical aging in the Landmark Direction and the Perspective Taking Tasks. We found no differences between groups in the Landmark Sequence Task. Given that participants were able to recall routes after training, these results suggest that typical and early signs of atypical aging result in differential memory deficits for aspects of route knowledge.

## Introduction

Declines in navigation abilities in both typical and atypical aging are now well established in a variety of tasks (Bellassen, Iglo, Cruz de Souza, Dubois, & Rondi-Reig, 2012; Head & Isom, 2010; Monacelli, Cushman, Kavcic, & Duffy, 2003; Zhong & Moffat, 2016). The vast majority of studies investigating the effects of (a)typical aging on navigation skills focus on a participant’s ability to learn unfamiliar routes or novel environments (Cherrier, Mendez, & Perryman, 2001; Cushman, Stein, & Duffy, 2008; Pengas et al., 2010). So far, our understanding of whether, and how, spatial representations differ in young and older participants after the successful learning of a novel route is limited. Here we address this question by using a novel route learning paradigm: our participants first learned short routes until they could successfully repeat them. To investigate how route representations are affected by typical aging and early signs of atypical aging, we then tested participants on various aspects of route knowledge.

Age-related declines in navigation abilities are most pronounced in hippocampal-dependent spatial tasks, i.e., tasks that require allocentric processing or cognitive map-like representations (Harris & Wolbers, 2013; Moffat, 2009; Wiener, de Condappa, Harris, & Wolbers, 2013). These differences in both typical and atypical aging are often explained by neurodegeneration of the hippocampus, one of the earliest brain areas affected in both healthy aging (Raz, Ghisletta, Rodrigue, Kennedy, & Lindenberger, 2010) and in atypical aging such as Alzheimer disease (AD) (deIpolyi, Rankin, Mucke, Miller, & Gorno-Tempini, 2007; Hort et al., 2007). (A)typical aging, however, also affects other navigation tasks that can be solved using egocentric navigation strategies, such as learning an unfamiliar route that is often conceptualized as learning a series of associative cues or recognition-triggered responses (“Turn left at the church,” Waller & Lippa, 2007).

Healthy older adults generally take longer to learn routes (Barrash, 1994) and perform significantly worse on a range of landmark-based tasks than young adults (Head & Isom, 2010). In experiments where older adults received the same amount of training as the young participants, older adults show impaired performance in locating where objects were encountered along the route (Gyselink et al., 2013) and in stating the sequence in which the objects were encountered (Head & Isom, 2010; Wiener , Kmecova, & de Condappa, 2012; Wilkiniss et al., 1997). Older adults also show impaired object-direction binding, i.e., they have less accurate knowledge of the direction in which the route continued at particular landmarks (Head & Isom, 2010; Wiener et al., 2012; Zhong & Moffat, 2016) and tend to point out salient objects rather than turns or intersections as being navigationally relevant (Lipman, 1991).

Declines in navigation performance become more pronounced if the older individual additionally develops a form of dementia, specifically amnestic Mild Cognitive Impairment (MCI) or AD (Benke, Karner, Petermichl, Prantner, & Kemmler, 2014; Cherrier et al., 2001; Cushman et al., 2008; deIpolyi et al., 2007; Monacelli et al. 2003; Pengas et al., 2010). Cherrier et al., (2001) compared typically aging adults and people with early AD in a series of route learning tasks. In contrast to healthy older adults, participants with AD showed profound difficulties in identifying a recently navigated route from a map perspective. Also, older adults with MCI and AD made significantly more errors than the typically aging adults when following a route without assistance and additionally misidentified the landmarks along the route (Benke et al., 2014). These results highlight the steep decline in navigation abilities associated with atypical aging.

While the studies reviewed above clearly demonstrate age-related declines in route learning abilities, both in typically and atypically aging adults, very little is known about how route memory is affected after the successful learning of a route. This is because different participant groups usually undergo the same training protocol, i.e., they are exposed to the route either for the same amount of time or are presented with the same number of training trials before route knowledge is assessed (Benke et al., 2014; Cherrier et al., 2001; Pengas et al., 2010). Assuming slower route learning in older adults (Head & Isom, 2010), they would have learned less about the route than the young participant group when they entered the test phase. While this approach is perfectly suited to study age-related differences in route learning, it may not be sensitive to highlighting differences in the content, format, and structure of route knowledge that is sufficiently detailed to support successful navigation of the learned route. Any differences in route knowledge can therefore either result from aging-related shifts in learning strategy, or could reflect different rates of knowledge acquisition between groups. In other words, it is not clear whether differences in knowledge about the order in which landmarks were encountered (Bellassen et al., 2012), or differences in identifying movement directions associated with landmarks (Head & Isom, 2010; Wiener et al., 2012) highlight specific age-related navigation deficits or instead reflect differences in general route knowledge resulting from slower learning.

As earlier studies have reported age-related strategy shifts in spatial learning (Rodgers, Sindone, & Moffat, 2012; Wiener, de Condappa, Harris, & Wolbers, 2013), it is conceivable that such strategy differences could result in (at least partly) different route representations that are best tested after routes have been learned. Aging also affects executive functions (Fjell, Sneve, Grydeland, Storsve, & Walhovd, 2017; Lezak, 1995), which may in turn affect people’s ability to learn different route representations simultaneously or switch between these representations during learning and/or recall. This again would be best assessed after routes have been learned successfully. Finally, age-related differences in memory decay are often not controlled for, which is problematic as forgetting could affect performance in tests of route knowledge that are administered after route learning.

To address these issues, we present a novel paradigm in which participants learned short novel routes through virtual environments. After successful learning, they were then confronted with several tasks assessing different aspects of route knowledge. The tasks we selected have been adopted from previous experiments that have addressed the effects of typical and atypical aging on route learning (Benke et al., 2014; Cherrier et al., 2001; Cushman et al., 2008) and assess knowledge of landmark sequence, sequence of direction changes, landmark-direction associations, as well as participants’ ability to recognize the learned route from map-like schematic drawings. After completing these tasks, participants were then asked to navigate the route again, which allowed us to control for potential effects of differential memory decay. To address the effects typical aging as well as the effects of early atypical aging, we tested a young participant group and two older participant groups, one of which scored high and the other scored lower on a neuropsychological screening tool for MCI.

Based on the literature discussed, we expected that our typically aging participants would perform generally worse than our younger participant group, and that our older participant group who showed early signs of atypical aging would perform generally worse than our healthy aging older participant group. Having said this, few, if any, studies so far addressed route knowledge after successful route learning. It is therefore possible that all participant groups perform similarly well in those tasks that capture the route knowledge that is particularly relevant during navigation. Given that route knowledge is often thought of as a series of stimulus-response associations (Waller & Lippa, 2007), knowledge about movement directions associated with landmarks or knowledge about the sequence in which landmarks are encountered are such candidates. In contrast, we expected effects of typical, as well as atypical, aging in the map-based task that required mental transformation between the egocentric route perspective and the map perspective, a process that is known to be affected by (a)typical aging (Cushman et al., 2008).

## Methods

### Participants

Sixteen young (mean age = 21.62 years, SD = 3.27; age range = 18–29 years; eight males and eight females) (*Young* group) and 33 older adults aged 65 years and over (American Psychological Association, 2014) took part in this study. All participants were recruited either through the Bournemouth University’s participant recruitment system or through opportunity sampling in the community.

### Older participant group

All older participants completed the Montreal Cognitive Assessment (MoCA), a 30-point test designed to test for healthy aging and to detect MCI and early-stage AD (Nasreddine et al., 2005). This screening tool has been shown to be highly sensitive in detecting early changes in cognition. Moreover, test scores correlate with the severity of cognitive impairment and AD (Freitas, Simões, Alves, & Santana, 2013). The most commonly used and accepted MoCA cut-off for healthy aging is 26/30. Lower scores indicate early atypical aging (Nasreddine et al., 2005). Interestingly though, some studies suggested that cut-offs as low as 22/30 (Lee et al., 2008) and 23/30 (Luis, Keegan, & Mullan, 2009) would also be suitable to separate healthy aging from atypical aging (see Julayanont, Phillips, Chertkow & Nasreddine, 2012, for a review). Here we used the suggested higher and lower MoCA cut-offs to split our older participants into two groups. Specifically, participants in the *Old High MoCA* group scored between 26 and 30 points and participants in the *Old Low MoCA* group scored between 22 and 25 points. Given that spatial disorientation and declines in navigation abilities are among the earliest symptoms of atypical aging and early mild cognitive impairments (Pai & Jacobs, 2004), we expected to find differences in navigation performance between the *Old High MoCA* group and the *Old Low MoCA* group if MoCA scores below 26 were, in fact, indicative of early atypical aging*.*

We had 17 participants in the *Old High MoCA* group (mean age = 70.06 years, SD = 7.04 years; age range = 65–83 years; 12 females and five males), whilst we had 16 participants in the *Old Low MoCA* group (mean age = 76.68 years, SD = 6.29 years; age range = 66–93 years; nine females and seven males). One participant scored below the suggested threshold of 22/30 (Lee et al., 2008; Luis et al., 2009). Their data were therefore not included in the final data set. Participants in the *Old High MoCA* group spent 11.73 years (SD = 2.75) in education and participants in the *Old Low MoCA* group spent 13.47 years in education (SD = 2.67). There was no significant difference between the *Old High MoCA* and the *Old Low MoCA* groups in terms of levels of education (t(31)=1.474, p=0.151). Following Nasreddine et al. (2005) criteria, all participants who had less than 13 years of education received an extra point to compensate for the effects of education on the test.

### Ethics

Ethical approval for the experiment was obtained from the Bournemouth University ethics panel. The researcher was present throughout the whole experiment, adopting a person-centered approach (Cowdell, 2006) to reduce any possible feelings of discomfort or stress (Dewing, 2008).

### Materials

#### The virtual environments

Using Vizard 3.0 (WorldViz) we created 12 different short virtual routes. Each route consisted of four four-way intersections and each route featured one left turn, one right turn, and one straight and one additional right turn, left turn, or straight movement. Each intersection could be identified by a unique object (landmark) mapped onto a cube that was suspended from the ceiling in the centre of the intersection. All 12 routes were created from the same environment, but each route featured a unique set of four landmarks (i.e., the same landmarks did not appear twice throughout the experiment) and consisted of a different sequence of turns. We created a video of each route that showed a passive transportation along the entire route (each video lasted 28 s). During the experiment, the videos were presented on a Toshiba Satellite Pro Laptop (22-in. screen).

### Procedure

Participants were first required to read the information sheet, to sign the consent form, and were then asked to fill out a brief participant information sheet to collect demographic information (age, gender, and years in education). After this, the older participants proceeded to complete the MoCA test, whereas the *Young* group started with the experiment.

Before beginning with the actual experiment, participants were shown a demo route and were talked through each of the tasks to ensure that they understood the procedure.

## Experiment

The experiment consisted of 12 separate blocks, each composed of a training phase, a test phase, and a route recall phase. Participants learned a different route in each block and the order in which routes were presented was random. Each block took approximately six min to complete, and participants were free to take breaks between trials if they wished.

### Training phase

**Fig. 1:**
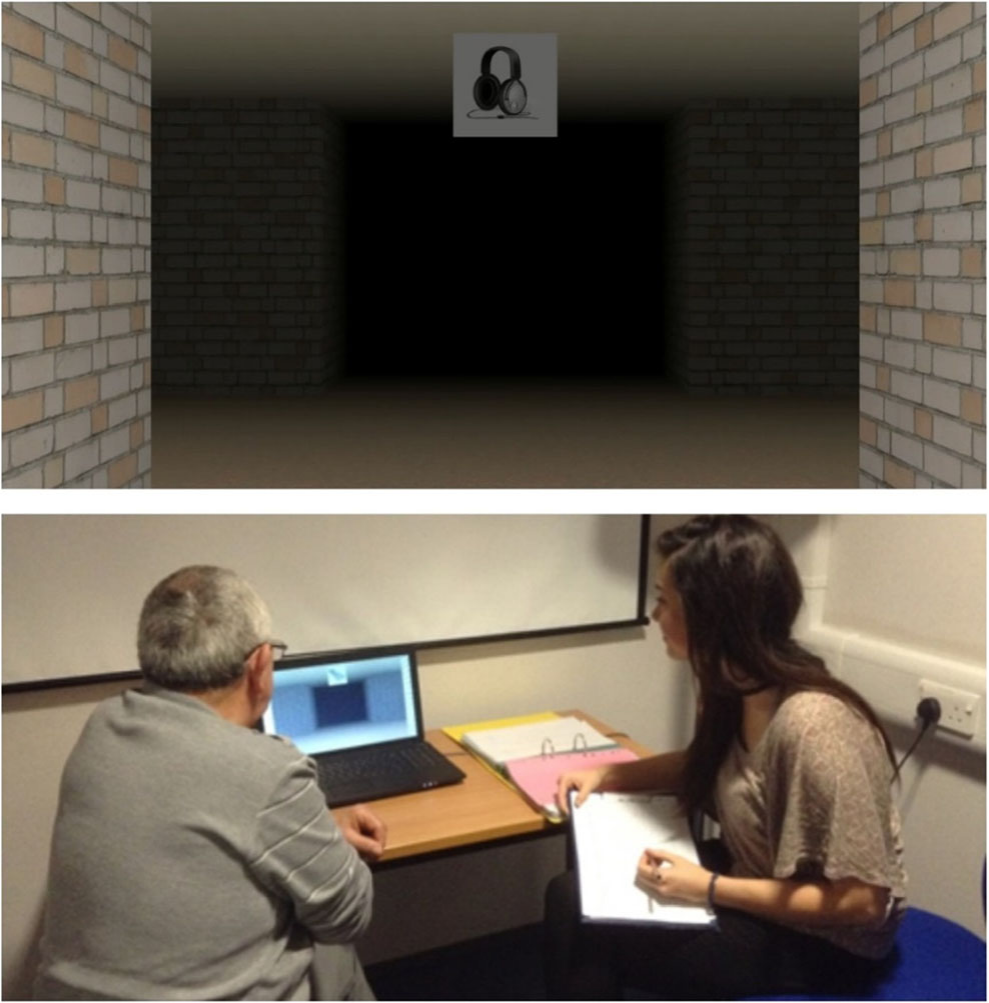
The top image shows the viewpoint of one of the routes used during the training phase. The lower image shows the testing room and room set-up during the experiment

In the training phase, participants first watched a video of a route (see Fig. 1). After the first presentation, participants were shown the route again, though this time the video was stopped at each intersection and participants were asked to indicate the direction of turn to continue along the route. If they made an error, they were shown the route again and asked for the directions of turn at each intersection. This procedure was repeated until participants were able to accurately indicate the direction of turn at each of the intersections. The number of errors and learning trials required to learn the route were recorded. Once participants successfully learned the route, they moved on to the test phase.

#### Test phase

**Fig. 2:**
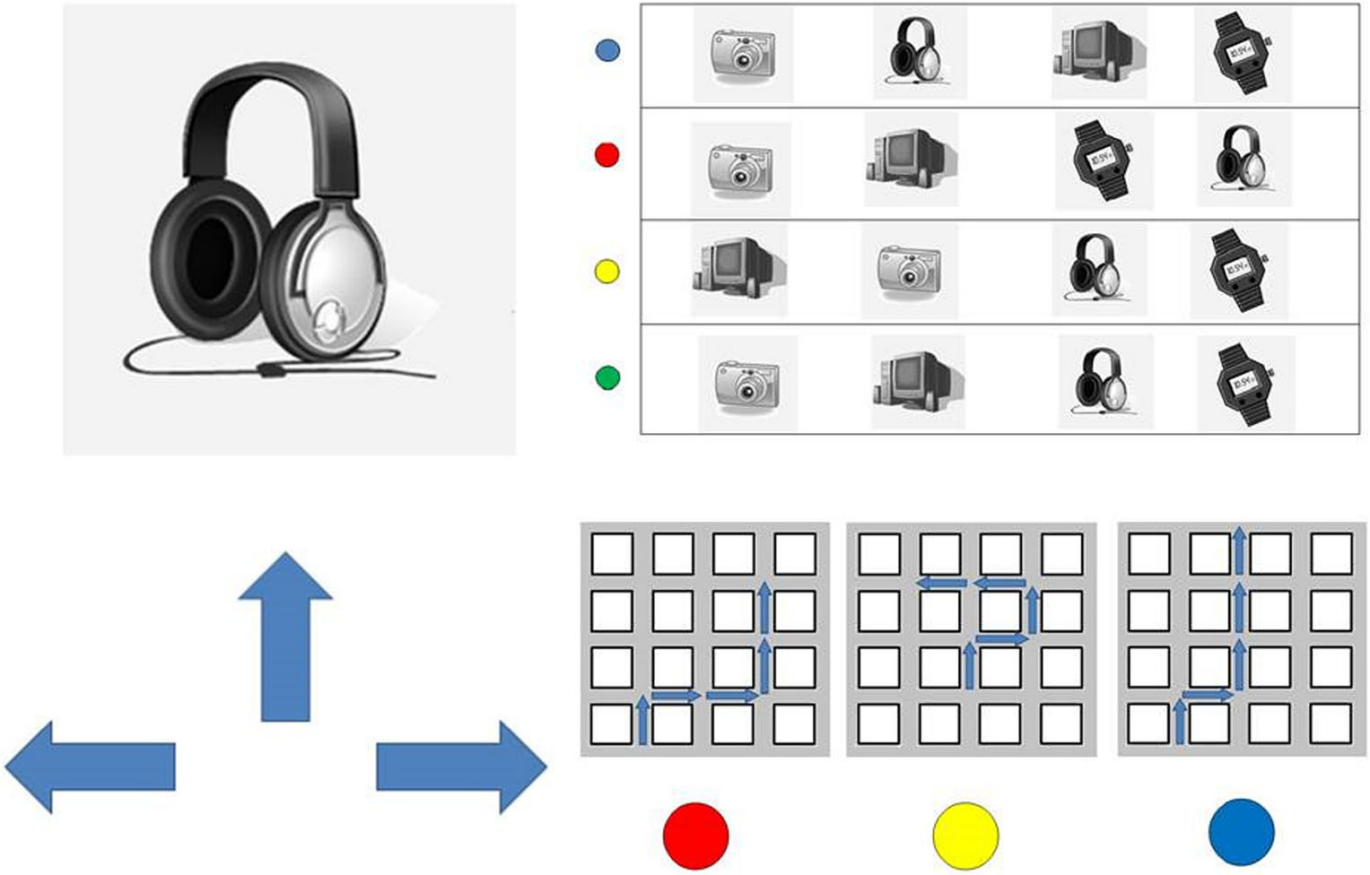
Stimuli used during the test phase. Left shows the ‘Landmark Direction Task,’ upper right shows the ‘Landmark Sequence Task,’ and the lower right shows the ‘Perspective Taking Task’

The test phase consisted of four different tasks that assessed different aspects of route knowledge:

Landmark Direction Task: Participants were presented with pictures (printed on A4 paper, see Fig. 2 for an example stimulus) of the landmark objects of the route one at a time and in randomized order. Their task was to indicate in which direction the route continued at the corresponding intersection. The Landmark Direction Task (sometimes also referred to as the associative cue task) required participants to associate a movement direction to the landmarks during route learning. We analyzed whether participants could or could not correctly recall the directions for all fours landmarks along a route. For each route participants’ responses were coded as correct or incorrect. Chance level for reporting all four directions correctly was 1.23 %.

**Fig. 3:**
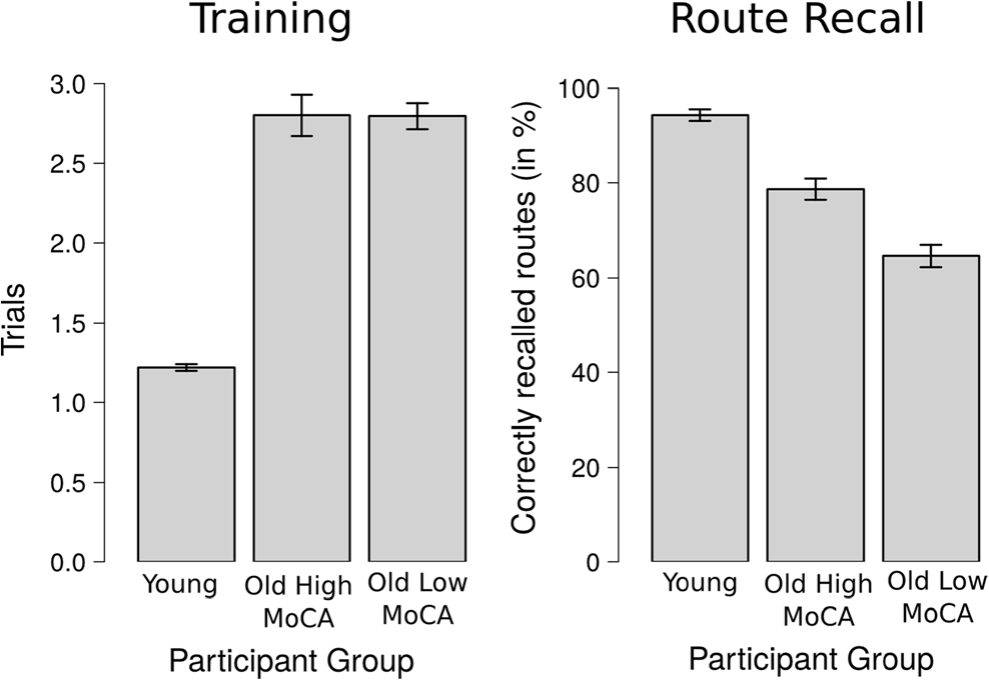
Left: learning performance (number of trials to watch the route) during the training phase for the *Young* group, the *Old High MoCA* group, and the *Old Low MoCA* group; right: recall performance (%) for the *Young* group, the *Old High MoCA* group, and the *Old Low MoCA* group

**Fig. 4:**
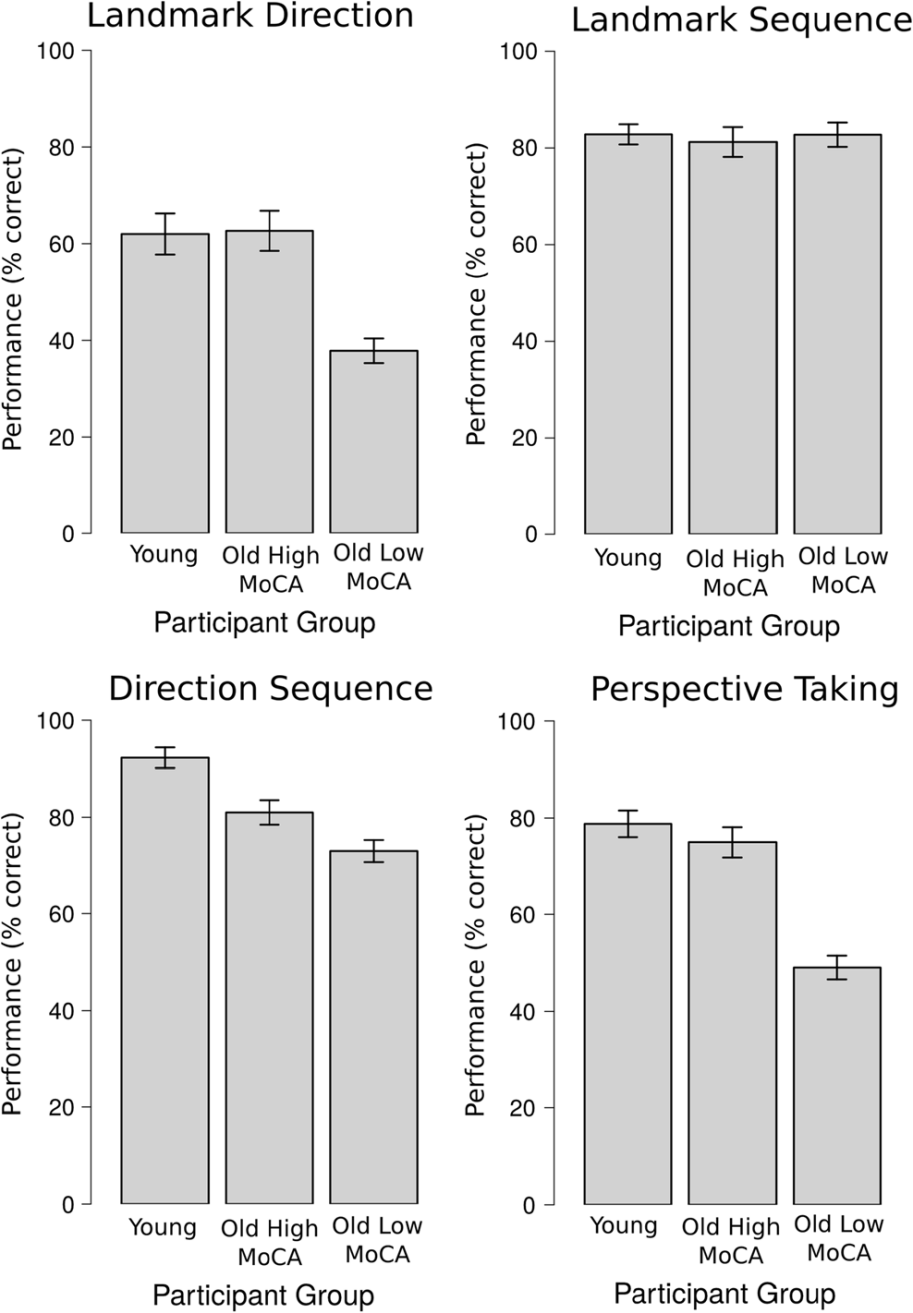
Upper left: Landmark Direction Task performance for the *Young* group, the *Old High MoCA* group, and the *Old Low MoCA* group. Lower left: Direction Sequence Task performance for the *Young* group, the *Old High MoCA* group, and the *Old Low MoCA* group. Upper right: Landmark Sequence Task performance (%) for the *Young* group, the *Old High MoCA* group, and the *Old Low MoCA* group. Lower right: Perspective Taking Task performance (%) for the *Young* group, the *Old High MoCA* group, and the *Old Low MoCA* group.

Landmark Sequence Task: Participants were presented with four different arrangements of the four landmark objects of the route printed on an A4 sheet of paper (see Fig 2 for an example stimulus). One of the arrangements displayed the correct temporal order in which the landmarks were encountered along a route, the other three arrangements were variations of the correct order (e.g., the second and third objects were swapped). Participants’ task was to indicate which row of landmarks displayed the correct order of landmarks from start to finish on the route. For each route, participants’ responses were coded as correct or incorrect. Given four possible choices, chance level for this task was 25 %.

Direction Sequence Task: In the Direction Sequence Task, participants were asked to verbally report the sequence of direction changes or movements along the route (e.g., “left, right, straight, right”). We analyzed whether participants could or could not correctly recall all four direction changes along a route. For each route participants’ responses were coded as correct or incorrect. Chance level for reporting all four direction changes correctly was 1.23 %.

Perspective Taking Task: Participants were presented with three different schematic map-like drawings of routes through a regular grid like environment (see Fig. 2 for an example stimulus). One of these schematized routes depicted the route they had just learned, while the other schematized routes were variations of the correct route (e.g., one turn was mirrored). The routes were printed on a sheet of A4 paper. Participants’ task was to indicate which route depicts the route they have just learned. The Perspective Taking Task required participants to recognize the route from a top-down map-like perspective. For each route, participants’ responses were coded as correct or incorrect. Given three possible choices, chance level for this task was 33.3 %.

#### Route recall phase

Once participants had completed the Test Phase, they were again presented with the video of the route to test whether they could still accurately recall the route. This was done to ensure that potential differences in Test Phase performance were not due to general memory decay. As in the learning phase, the video was stopped at each of the four intersections along the route, and participants were required to state the correct direction at each intersection.

### Task order in the test phase

The tasks in the test phase were presented in two different orders. Order 1 was: Perspective Taking, Direction Sequence, Landmark Sequence and Landmark Direction. Order 2 was: Landmark Sequence, Perspective Taking, Direction Sequence and Landmark Direction. After six routes, the order switched from Order 1 to Order 2 or vice versa (counterbalanced between participants).

### Analysis

To investigate the effects of typical and early atypical aging on performance we ran linear mixed effect (LME) models for accuracy for each of the tasks (using R and the lme4 package; Bates, Maechler, Bolker, & Walker, 2014). We defined two a priori contrasts: First, between the *Young* group and the *Old High MoCA* group to study the effect of typical aging, and second, between the *Old High MoCA* group and *Old Low MoCA* group to study the effect of early atypical aging.

The analysis included participant group as a fixed effect with the individual participants and the routes as random intercepts. We report the coefficient and standard error estimates (SE) and interpreted effects with a value of *t > 2* as reliable, although we also report estimated p values.

## Results

### Learning and recall

The analysis of route learning and route recall performance encompassed all 12 trials per participants. See Fig. 3 for learning performance and route recall performance for each of the three participant groups.

#### Training phase

On average the *Young* participants viewed routes 1.22 times during the training phase, whilst both the *Old High MoCA* group and the *Old Low MoCA* group viewed the routes 2.80 times. The LME analysis revealed significant differences between the *Young* and *Old High MoCA* (typically aging) participant group (b = 1.58, SE = 0.22, t = 7.15), but no differences between both the two older participant groups (b = -0.01, SE = 0.22, t = -0.02).

#### Route recall

On average the *Young* participants recalled 94.3 % of the routes correctly, the *Old High MoCA* group recalled 78.7 % of the routes correctly, and the *Old Low MoCA* group recalled 64.6 % of the route correctly. The LME analysis revealed significant differences for both a priori contrast, i.e., between the *Young* and *Old High MoCA* participants (b = -1.54, SE = 0.40, z = -3.82, p < 0.001) and between the two older participant groups (b = -0.74, SE = 0.30, z = -2.51, p = 0.01).

While *Young* participants showed better performance in both training phase and route recall, these results demonstrate a dissociation between learning and recall in our older participant groups. Specifically, the route learning during the training phase was not affected in the *Old Low MoCA* group, while route recall was affected.

### Association between acquisition and forgetting

To investigate whether the number of learning trials had an influence on recall performance, we compared the number of training trials for correctly and incorrectly recalled routes for each of the participant groups. None of these comparisons were statistically significant, suggesting that there was no association between acquisition and forgetting in this study (note that the majority of the younger participants did recall all routes correctly, so that we could run this analysis only on a subset of the younger participants: *Young* t(6) = 0.379, p = 0.718; *Old High MoCA* t(15) = -0.103, p = 0.909; *Old Low MoCA* t(15) = 0.346, p = 0.734).

### Test tasks

The analysis of the four test tasks only included the data from routes that were correctly recalled after the test phase. See Fig. 4 for performance for each of the four test tasks for each of the participant groups.

#### Landmark direction task

On average, *Young* participants remembered the directions for all landmarks for 62.00 % of the routes, the typically aging group achieved similar scores (62.55 %), while the *Old Low MoCA* group remembered the directions for all landmarks for only 37.84 % of the routes. An LME did not reveal significant differences between the *Young* and *Old High MoCA* group (b = -0.10, SE = 0.46, z = -0.21, p = 0.83). The comparison between the two older participant groups, however, was significant (b = -1.28, SE = 0.47, z = -2.68, p < 0.01).

#### Landmark sequence task

Performance in remembering the sequence in which the four landmarks were encountered along the route were very similar between participants, with 82.81 % accuracy in the *Young* participant group, 81.23 % accuracy in the *Old High MoCA* group, and 82.74 % accuracy in the *Old High MoCA* group. An LME did not reveal significant differences between the *Young* and *Old High MoCA* group (b = -0.04, SE = 0.43, z = -0.09, p = 0.93), or between the *Old High MoCA* group and *Old Low MoCA* group (b = 0.15, SE = 0.46, z = 0.33, p = 0.75).

#### Direction sequence task

On average, *Young* participants successfully remembered the sequence of direction changes for 92.26 % of the routes, our *Old High MoCA* group achieved 81.01 %, while our *Old Low MoCA* group remembered the sequence of direction changes for 72.93 % of the routes. An LME did reveal significant differences between the *Young* and *Old High MoCA* group (b = -1.14, SE = 0.49, z = -2.36, p = 0.02). The comparison between the two older participant groups, however, did not reveal a statistically significant difference (b = -0.52, SE = 0.43, z = -1.21,p = 0.23).

#### Perspective taking task

On average, *Young* participants chose the correct map in 78.8 % of the trials, the *Old High MoCA* group in 74.8 % of the trials, and the *Old Low MoCA* group recalled 49.0 % of the trials. An LME did not reveal significant differences between the *Young* and *Old High MoCA* group (b = -0.17, SE = 0.38, z = -0.43, p = 0.67). The comparison between the two older participant groups, however, was significant (b = -1.44, SE = 0.39, z = -3.74, p < 0.001).

#### Performance over the course of the experiment

Learning several routes in similar environments could lead to interference which could result in declining performance over the course of the experiment. To test whether performance was affected by interference we calculated correlations between our different measures of route learning and knowledge (Route Learning, Route Recall, Direction Sequence Task, Perspective Taking Task) and the block of the experiment (1–12) for each participant. Only two correlations were significant: for the Landmark Sequence Task for the *Old High MoCA* group (r = .671, n = 12, p = .017) and for the Landmark Direction Task for the *Young* group (r = .660, n = 12, p = .019). Note that both correlations were positive, suggesting increasing performance over the course of the experiment. These results suggest that interference was not an issue in this study.

### Controlling for age

As mentioned in the participants section, participants in the *Old Low MoCA* group were older than participants in the *Old High MoCA* group (t(31)=-3.027, p<.005 t(31) = -3.027, p < 0.005). It could therefore be argued that this age difference, rather than differences in cognitive abilities as assessed by the MoCA between the two older groups, explains the described effects. To test this, we matched pairs of older participants based on their MoCA score. We then assigned the older participant of the pair to the older participant group (“Old-Old group”) and the younger of the pair to the younger participant group (“Old-Young group”). We could match 28 out of the 33 participants (in cases in which there was an unequal number of participants with the same MoCA score, we were left with one participant – the one in the middle - who could not be matched). By matching participants in this way, we created two participant groups that were perfectly matched on MoCA score, but that differed in age (mean age Old-Young group: 68.43, SD = 3.87; mean age Old-Old group: 77.93, SD = 7.63, t(26) = -4.153, p < 0.001). We then compared performance in the different tasks between these groups. The results are presented in Table 2. Importantly, none of the comparisons revealed a significant difference between the Old-Old and the Young-Old group. This suggests that declines in cognitive abilities rather than differences in age between the two older participant groups drove the effects we reported in the original analyses above.

## Discussion

In this study, we presented a novel route learning paradigm to investigate how aging affects route knowledge after successful learning novel short routes. To do so, participants were first trained until they could replicate the routes without errors. In the subsequent test phase, they were then asked to complete several tests assessing their knowledge of the route. Afterwards, participants were asked to recall the route once more to ensure any differences between groups during the test phase did not results from different rates of memory decay.

Comparing performance between our *Young* participants and the *Old High MoCA* group scores allowed us to investigate the effects of healthy aging on route learning and memory. By comparing performance between the *Old High MoCA* and the *Old Low MoCA* groups, we aimed to investigate the effect of early signs of atypical aging on route learning and memory. The findings highlight clear differences between the two older groups in a number of tasks. This supports the argument that (1) the MoCA is a sensitive measure to screen for early atypical aging (Nasreddine et al., 2005) and (2) that declines in navigation abilities are among the earliest sign of atypical aging (Pengas et al., 2010). In the following, we therefore discuss differences between the two older participant groups in context of early atypical aging.

Before discussing the results in more detail, it is important to highlight that the result pattern of the six tasks was complex. We found significant effects of typical as well as early atypical aging in only one task (Route Recall Task). We found effects of typical aging but not of early atypical aging in two tasks (Training Phase and Direction Sequence Task), and effects of early atypical aging but not of typical aging in two other tasks (Landmark Direction Task and Perspective Taking Task). Finally, one task (Landmark Sequence Task) was neither affected by typical nor by early atypical aging (for an overview, see Table 1). This heterogeneous result pattern strongly suggests that declining navigation abilities in both typical and early atypical aging are not the result of general declines in learning and memory abilities. Rather (a)typical aging affects specific mechanisms and components of navigation.

**Table 1:** Summary of the results per task, which highlights the effects of typical aging (comparisons between *Young* and *Old High MoCA* group) and the effects of early atypical aging (comparisons between *Old High MoCA* group and *Old Low MoCA* group)

Task	*Young* group vs. *Old High MoCA* group (typical aging)	*Old High* vs. *Old Low MoCA* group (early atypical aging)
Training Phase	Yes	No
Route Recall	Yes	Yes
Landmark Direction Task	No	Yes
Landmark Sequence Task	No	No
Direction Sequence Task	Yes	No
Perspective Taking Task	No	Yes

**Table 2:** Results of t-test comparisons between the Old-Young and Old-Old groups, who were matched specifically on MoCA score (degrees of freedom = 26). The results show that none of the comparisons were statistically significant; only one task, the Training Phase, was close to being significant. We take this as strong evidence that the differences in performance between the two older participant groups in our original analysis resulted from differences in cognitive abilities (as assessed by the MoCA) and not from differences in age

Condition and Task	Old-Young Mean	Old-Young SD	Old-Old Mean	Old-Old SD	T	P value
Training Phase	2.54	0.71	3.11	0.87	-1.895	0.07
Route Recall	75.60	12.43	67.26	20.53	1.299	0.21
Landmark Direction	72.19	18.37	76.49	15.13	-0.676	0.51
Landmark Sequence	79.98	17.92	78.96	21.59	0.14	0.89
Direction Sequence	91.27	7.96	85.63	13.22	1.368	0.18
Perspective Taking	63.72	19.92	58.97	27.00	0.53	0.60

### Training phase and route recall

In line with earlier research (Head & Isom, 2010), older participants showed slower rates of route learning, requiring more than twice as many training trials to learn the routes as compared to the *Young* participant group. While the two older participant groups did not differ in their learning performance, they differed in their recall performance. Specifically, the *Old Low MoCA* group recalled less than 2/3 of the routes after the test phase. In fact, performance in the route recall task differed between all three participant groups with the *Young* group showing best performance followed by the *Old High MoCA* group and then the *Old Low MoCA* group. While forgetting is rarely studied in the context of spatial cognition and navigation, it has been studied in other cognitive domains and accelerated forgetting has been associated with both healthy aging (Huppert & Kopelman, 1989) and with mild cognitive impairments (Geurts, van der Werf, & Kessels, 2015; Walsh et al., 2014). Taken together, these results suggest (1) that typical aging is associated with slower route learning, (2) that early atypical aging does not affect route learning, and (3) that aging as well as early atypical aging are associated with faster forgetting of route knowledge. While these findings are in line with earlier research, we did not find that slower rate of acquisition or learning to criterion was associated with accelerated forgetting in our study (Macdonald, Stigsdotter-Neely, Derwinger, & Bäckman, 2006).

Given the different rates of forgetting between participant groups, we included only data in the test phase analyses from routes that participants recalled correctly. This ensured that participants still knew the routes in the actual test phase, and any group differences in that phase therefore did not result from different rates of forgetting.

### Test phase

Interestingly, we did not find any differences between groups in the Landmark Sequence Task. We did not find differences between our *Young* participants and the typically aging adults in the Perspective Taking Task and Landmark Direction Task, but we found differences in the Direction Sequence task. Comparisons between the two older participant groups revealed significant differences for the Perspective Taking and the Landmark Direction tasks, but not for the Landmark Sequence or Direction Sequence tasks.

Some of these results are surprising at first glance given that several earlier studies reported that typical aging was associated with declines in perspective taking abilities (De Beni, Pazzaglia, & Gardini, 2006; Puglisi & Morrell, 1986), declines in the knowledge of the sequence in which landmarks were encountered during route learning (Bellassen et al., 2012; Head & Isom, 2010; Wiener et al., 2012), and declines in ability to bind directional knowledge to landmarks (Head & Isom, 2010; Wiener et al., 2012). It is likely that these differences between our study and these earlier studies can be explained by the fact that we tested participants’ route knowledge only after they had successfully learned the routes, which took our older participant groups twice as long to learn as the *Young* group. In other words, our older participant groups had more exposure to the routes, which was – at least for the higher MoCA group – sufficient to encode the route knowledge required to solve the test phase tasks.

The performance differences between the two older participant groups in the Perspective Taking and the Landmark Direction tasks are in line with earlier studies: Cherrier et al. (2001) used a task similar to our Perspective Taking Task and found significant differences between healthy older adults and those with AD. Similarly, Cushman et al. (2008) found that participants with MCI and early AD had particular problems when asked to indicate the positions at which they encountered landmarks along a route in a schematic drawing of the route (see also deIpolyi et al., 2007). Recognizing a route, experienced and encoded in an egocentric reference frame, from a map, requires either the construction of an allocentric representation or a mental transformation. Both of these processes have been closely associated with the hippocampal circuit (King, Burgess, Hartley, Vargha-Khadem, & O'Keefe, 2002), an area that is among the earliest affected by MCI and AD (Fjell, McEvoy, Holland, Dale, & Walhovd, 2013; Raz et al., 2010). The performance differences between the older participant groups in the Landmark Direction Task, which is essentially an associative learning task assessing people’s ability to bind directional information to specific landmark object, is not surprising as earlier studies highlighted impaired associative learning in early atypical aging (Boespflug, Eliassen, Welge, & Krikorian, 2014).

Surprisingly, we did not find performance differences between the two older participant groups in the Landmark Sequence or the Direction Sequence tasks. Earlier navigation studies have described that the learning of sequences of turns relies on the hippocampal circuit (Igloi, Doeller, Berthoz, Rondi-Reig, & Burgess, 2010), which undergoes substantial functional and structural changes during the (a)typical aging process (Fjell et al., 2013). Moreover, earlier studies explicitly demonstrated that people with MCI and AD have profound deficits in ordering objects encountered along a route (deIpolyi et al., 2007) and when learning a sequence of direction changes (Bellassen et al., 2012).

We believe that these differences in the paradigms used to measure sequence and order memory may explain these differences between our and earlier studies (e.g., Monacelli et al., 2003; Pengas et al., 2010). Specifically, we used relatively short routes, assessed route knowledge only after participants successfully learned the routes and we focused on routes that participants were able to correctly recall later in order to investigate the effects of (a)typical aging on the content of route knowledge rather than on route learning performance.

Further studies are needed to investigate the impact of these methodological differences on route learning and route knowledge in more detail. It would, for example, be important to investigate how the different aspects of route knowledge (sequence knowledge, associative cue knowledge, etc.) develop as people learn to navigate the route. Moreover, our Landmark Direction and the Landmark Sequence Tasks are cued recall tasks, while the Direction Sequence Task represents a free recall task. Earlier learning studies using non-spatial stimulus material demonstrated that free recall is more strongly affected by early atypical aging than cued recall (Grober & Buschke, 1987; Grober, Veroff, & Lipton, 2016). Further research is needed to understand the effects that (a)typical aging has on free and cued recall in the context of navigation and route learning in particular. To develop a better understanding of the variability in route learning between groups, future studies should also consider individual difference in spatial abilities such as visuo-spatial working memory or mental rotation which have been suggested to be closely related to age-related differences in navigation abilities (Gyselinck et al., 2013).

When comparing overall performance levels of the test phase tasks, it is striking that performance was very good in the Direction Sequence Task (over 90 % correct in the *Young* participant group). In contrast, performance in the Landmark Direction Task, often used as a measure of route knowledge in other studies (Head & Isom, 2010; Mallot & Gillner, 2000; Waller & Lippa, 2007; Wiener et al., 2013), was considerably lower. Given that participants were able to recall the learned routes shortly after completing these tasks, these results suggest that participants primarily relied on memorizing sequences of turns rather than using an associative cue strategy (Waller & Lippa, 2007). This could be due to the fact that we used short routes with only four decision points in this study. Note however, that none of the route knowledge tasks in the test phase in isolation captured participants’ route knowledge perfectly. If that was the case, we expected participants to perform perfectly, reaching 100 % performance on at least one of the test phase tasks. This suggests either that (1) participants relied on the various aspects of route knowledge tested, (2) or that none of our tasks fully captured the information participants used to learn the routes.

It is also important to note that the *Old Low MoCA* group performed considerably worse than the *Young* and/or the *Old High MoCA* group in three of the four test phase tasks even though they were able to recall the learned routes in the subsequent route recall phase. This may suggest that they have used different strategies than the *Young* and *Old High MoCA* participants to memorize the routes and that these strategies are not captured well by our test phase tasks. Additionally, declining cognitive abilities may have forced the *Old Low MoCA* participants to focus all their efforts on learning and recalling the routes, which leaves fewer resources that could contribute to memorizing aspects of the route that were required to solve all the test phase tasks. While our current data does not allow us to test these explanations, we currently run further experiments to address these issues. Our study also has implications for other studies that use, or have used, the MoCA to screen for cognitive impairments. Some studies have suggested scoring criteria as low as 21/30 (Freitas et al., 2013) or 22/30 (Lee et al., 2008) for differentiating healthy aging from MCI. Here, we demonstrated that the participant group for which we used a cut-off of 22/30 has shown substantial deficits in several spatial tasks when compared to the participant group with the higher cut-off of 26/30 (Nasreddine et al., 2005). Studies that use the lower cut-off may have, as a result, overestimated the effects of typical aging (Harris & Wolbers, 2013; Wiener et al., 2013) and future studies addressing the effects of typical aging should use the more conservative higher cut-off of 26/30 (Nasreddine et al., 2005).

## Conclusion

In this study, we developed a new paradigm to investigate the effects of typical and early atypical aging on route learning performance and on route knowledge after successful learning of short unfamiliar routes. Results suggest that typical aging affected route learning performance, participants’ knowledge of the sequence of turns along the route as well as their ability to recall the routes later. Early signs of atypical aging did not affect route learning, but participants’ ability to recognize the route on a map, their ability to associate landmark to directions and their ability to recall the routes later. Importantly, differences between groups in the test phase did not reflect general age-related differences in learning rates or memory decay, as we only included data from routes that participants could successfully replicate later.
